# Validating the use of veterans affairs tobacco health factors for assessing change in smoking status: accuracy, availability, and approach

**DOI:** 10.1186/s12874-018-0501-2

**Published:** 2018-05-11

**Authors:** Anne C. Melzer, Erika A. Pinsker, Barbara Clothier, Siamak Noorbaloochi, Diana J. Burgess, Elisheva R. Danan, Steven S. Fu

**Affiliations:** 1 Center for Chronic Disease Outcomes Research, Minneapolis VA Health Care System, Veterans Drive, Minneapolis, MN 55417 USA; 2 Department of Medicine, University of Minnesota, Minneapolis, MN 55417 USA; 3 Pulmonary and Critical Care Medicine, Minneapolis VA Health Care System, Minneapolis, MN USA

**Keywords:** Tobacco cessation, Smoking, Behavior change, Methods, Electronic health record, Health services

## Abstract

**Background:**

Accurate smoking status is key for research purposes, but can be costly and difficult to measure. Within the Veteran’s Health Administration (VA), smoking status is recorded as part of routine care as “health factors” (HF)—fields that researchers can query through the electronic health record (EHR). Many researchers are interested in using these fields to track changes in smoking status over time, however the validity of this measure for assessing change is unknown. The primary goal of this project was to examine whether HFs can be used to accurately measure change in tobacco status over time, with secondary goals of assessing the optimum timeframe for assessment and variation in accuracy by site.

**Methods:**

Secondary analysis of the Veterans VICTORY study, a pragmatic smoking cessation randomized controlled trial conducted from 2009 to 2011. Eligible subjects were identified via the EHR using a past 90-day HF indicating current tobacco use (for example: “CURRENT SMOKER”, “CURRENTLY USES TOBACCO”). Participants were surveyed at 1 year to determine prolonged smoking abstinence. We identified HFs for tobacco status within +/− 120 days of the follow-up survey mailing date and recorded the temporally closest HF. Among subjects with both measures, we compared the two for agreement using kappa statistics and concordance.

**Results:**

1713 subjects (33%) had both follow-up survey and HF data, 1594 (31%) had only a survey response, 790 (15%) had only HF and 1026 (20%) had neither. For subjects with both measures, there was 90% concordance and moderate agreement (Kappa 0.48, 95%CI 0.41–0.55, Sensitivity 54.4, 95%CI 41.1–67.7, Specificity 94.3, 95%CI 87.5–100.0).

**Conclusions:**

We found high concordance but only moderate agreement by kappa statistics between HFs and survey data. The difference is likely accounted for by the natural history of quit attempts, in which patients cycle in and out of quit attempts. HFs appear to provide an accurate measure of population level quit behavior utilizing data collected in the course of clinical care.

**Electronic supplementary material:**

The online version of this article (10.1186/s12874-018-0501-2) contains supplementary material, which is available to authorized users.

## Background

Tobacco related illness remains one of the leading causes of preventable death nationally [1, 2], and is one of the top drivers of healthcare costs among military Veterans [3]. Because of the importance of addressing tobacco use in the clinical setting, smoking status is routinely recorded as a field in the electronic health record (EHR) [4, 5]. This has led to interest in utilizing these fields for research and evaluation purposes. Accurate smoking status is key for a number of research purposes, including the ability to assess the outcome of smoking cessation programs. Healthcare organizations such as the Veterans Health Administration (VA) or academic medical centers are a common setting to test tobacco cessation interventions [6–8], as they allow researchers to capitalize on contacts with high-risk patients that occur as part of routine care. Traditional research methods of capturing smoking status, which require at a minimum that the patient be contacted by phone or mail and have their reported information entered into a database, can be costly and time consuming. This potentially limits their utility for evaluating system-wide tobacco interventions, or tobacco interventions offered in the setting of clinical care. Despite the efforts of study staff, some patients will remain non-responders to surveys, which may bias the results. Finally, patient reports of tobacco status may also be subject to biases such as social desirability or stigma, which may differ between research and routine clinical settings. Computer-based approaches such as natural language processing require significant programmer expertise, and still require a large investment of time on the part of investigators at each institution to ensure accuracy [9]. Use of a validated, EHR-based method could streamline this process.

Within the VA EHR, tobacco use status is recorded in the form of Health Factors (HFs)—electronic data fields that are entered at the time of clinical care and can be readily retrieved. The VA remains the largest single healthcare system in the United States, with 144 hospitals and over 8 million patients [10]. Examples of common tobacco health factors include “CURRENT SMOKER”, “CURRENTLY USES TOBACCO” and “NON-TOBACCO USER.” Smoking status gathered by HF does not require additional resources, such as research staff, to obtain it. Because they are tagged data fields, similar to diagnostic codes, they can be easily retrieved. However, because they are entered by clinical staff and not study personnel, they are not subject to the more rigorous definitions of tobacco use or abstinence employed in research settings, making their use and limitations for research purposes less clear. For example, staff may erroneously identify a patient as a smoker who uses non-combustible tobacco products. HFs are updated frequently during hospitalizations, primary care visits, and mental health appointments, with providers prompted to enter them at least annually. Most VA facilities prompt nursing staff to enter HFs at least annually when a patient presents to the primary care clinic. These automatic prompts do not rely on each provider to remember to make the assessment, which increases the likelihood that HFs will capture changes in smoking status. A previous study compared past year HFs with baseline tobacco status obtained by survey in two cohorts including over 18,000 patients and found high agreement with survey data, particularly among never smokers and current smokers [11]. Subsequently, the VA performed a detailed examination of the utility of HFs for performing Health Services Research. The resulting technical report, produced by the VA Health Economic Resource Center (HERC), examined the records of 5.7 million unique Veterans from 2009 to 2011, and found that the majority of Veterans (70.3%) had a tobacco HF, with significant variation in availability by site. Of patients with an available baseline measure, almost 90% had a follow-up measure within 2 years, with an apparent quit rate of 12.3% among current smokers. The HERC technical report concluded that HFs provide a useful tool for epidemiologic studies and long-term follow-up on quit behaviors within the VA. A more recent analysis used these methods to assess over 700,000 current and 400,000 former smokers within the VA, and found similar rates of the availability of health factors, and an annual quit rate of approximately 12% [12]. However, while both the HERC report and the recent publication by Barnett et al. demonstrate conclusively that HFs are widely available and change over time, the authors were unable to assess the accuracy of the change in status, only that the change had occurred [13]. To date, no studies have reported the accuracy of HFs for assessing change in tobacco status over time by comparing them to another validated method.

Because of these two features—high baseline validity and the possibility of tracking changes in smoking status over time—HFs provide an attractive EHR-based means to capture changes in tobacco status in response to interventions. HFs could be particularly useful for evaluating large scale, multicenter or system-wide interventions that would be cost-prohibitive to assess by conventional means. EHR-based smoking status could also fill in results for survey nonrespondents. These missing data elements are commonly accounted for by statistical means [14], but HFs may prove to be more accurate. We aimed to examine the utility of HFs as an alternative method for obtaining follow-up data on smoking status using the EHR. Specifically, we assessed whether HFs can provide an accurate, cost and labor-saving means of assessing the response to tobacco interventions. To accomplish this goal, we performed a secondary analysis of a randomized controlled trial of proactive outreach for tobacco cessation, comparing the survey tobacco outcomes from the trial with HF data from the EHR. The primary goal of our analysis was to determine the proportion of subjects with a change in HF tobacco status, and assess the accuracy of this information in comparison to survey responses. Our secondary goals were to assess the optimum timeframe for obtaining follow-up HFs, and examine the variability in accuracy and availability of HF data by site. In addition, we performed a stratified analysis of patient groups with frequent healthcare contacts to see whether HFs were more accurate among these subgroups of participants.

## Methods

### Study design and participants

We performed a secondary analysis of the Veterans Victory Over Tobacco Study (VICTORY), a pragmatic trial of a proactive outreach, population-based intervention for tobacco treatment. A full report of the methods and the primary outcome results of the original randomized trial have previously been published [15]. In brief, subjects were recruited from four VA sites, selected to be nationally representative. Sites included: G.V. (Sonny) Montgomery VA Medical Center (Jackson, MS), James A. Haley VA Medical Center (Tampa, FL), Minneapolis VA Medical Center (Minneapolis, MN), and New York Harbor VA Medical Center (New York, NY). 6400 participants were enrolled from October 2009 to September 2010 until we reached the number of participants necessary to detect a 2% difference in population level cessation outcomes, and follow-up was completed in November 2011. Subjects were randomly selected Veterans aged 18 to 80 years who were identified as likely current smokers. As part of the pragmatic nature of the trial, subjects were screened for inclusion via a HF indicating tobacco use during a primary care visit within the prior 3 months. Smoking status was then confirmed by study personnel prior to enrollment. Exclusion criteria were minimal (ICD-9 diagnosis of dementia or severe persistent mental illness, 10 or more mental health clinic visits in the prior year, no valid contact information). Inclusion/exclusion criteria were applied using the VA HER.

In order to maintain balance within site, participants were randomized 1:1 to usual care versus the proactive outreach intervention, clustered within each site, and were enrolled regardless of current intention to quit smoking. Participants in the usual care arm could access any tobacco treatments recommended by their provider, including services through the VA or the state quitline. The proactive arm received both 1) proactive outreach (mailed invitation materials followed by telephone outreach) followed by 2) the choice of telephone or in-person cessation counselling. Phone counseling was provided by trained counselors located at the Minneapolis VA. Subjects who chose in-person counseling were connected with local VA cessation services, and all subjects were able to receive medications through their VA provider, which was facilitated by the study team. The project was approved by the Minneapolis VA Institutional Review Board (IRB) and the IRB of each study site and all studies were conducted in accordance with the ethical principles outlined in the Department of Veterans Affairs Good Clinical Practices. Data was stored according to the VA requirements for encryption, with access to identifiable data limited to the minimum number of required study personnel.

### Data collection and measures

Data was obtained via patient survey and the VA EHR. Survey data collection occurred by mail at baseline and at 12 months after randomization. Survey mailing staff were blind to the participants’ group assignment at the time of mailing. Smoking status was measured using self-reported 6-month prolonged smoking abstinence. The survey was developed in accordance with the recommendations for assessing smoking abstinence recommended by the Society for Research on Nicotine and Tobacco [16]. The study definition allowed for some lapses in cigarette smoking. Patients were categorized as not achieving 6-month prolonged abstinence if they indicated smoking in the past 30 days, had smoked at least once on seven consecutive days out of the past 6 months, or smoked at least once on two consecutive weekends. All analyses used self-reported 6-month prolonged abstinence as the conventional gold standard.

Administrative data, including demographics, clinical characteristics, and smoking status were obtained from the VA National Patient Care Databases. Demographic variables included age, gender and race. For individuals who had missing race on the baseline survey, race was filled in from administrative data in the EHR. Clinical characteristics were obtained from the EHR. We defined chronic lower respiratory disease (chronic bronchitis, chronic airway obstruction, emphysema, asthma, and bronchiectasis), mental illness (depression, post-traumatic stress disorder, anxiety, substance use disorder, serious mental illness), and cardiac disease using ICD-9 codes. We also used ICD-9 codes to calculate the Charlson comorbidity index, a validated administrative measure of the burden of disease, with higher scores indicating a greater burden [17].

We also measured smoking status using HFs. We extracted all tobacco-related HFs within 120 days before or after the 12-month follow-up survey mailing, therefore spanning a maximum time period of 240 days surrounding the date of mailing. HFs are generated when a member of the health care team enters a patient’s response into a specially templated form. These entries tag the note with a tobacco HF that, unlike a text entry in a progress note, is easily retrievable by electronic query. Nurses may be prompted to enter tobacco use information annually, at the time of a hospitalization, or change in clinical status.

All the resulting HFs were reviewed by the study team to assign the follow-up smoking status (current smoker or quitter). For example, a patient was identified as an ongoing smoker if the HF label contained information indicating: ‘CURRENT SMOKER’, ‘TOBACCO CURRENT USER’, ‘REFUSED SMOKING CESSATION’, ‘TOBACCO USER’, or similar. A patient was identified as a quitter if their follow-up tobacco status indicated ‘QUIT TOBACCO IN THE LAST 12 MONTHS’, ‘NON TOBACCO USER - QUIT IN PAST YEAR,’ ‘CURRENT NON-TOBACCO USER’ or similar. See Additional file 1 for complete definitions of tobacco use statuses. Patients whose HFs indicated use of a different form of tobacco, such as chewing tobacco, but not cigarettes were characterized as nonsmokers.

### Statistical analysis

Participants were grouped by source(s) of their follow-up smoking status and various baseline characteristics were compared by these four resulting groups (survey only, HF only, both, and neither) by using Pearson Chi-square tests for categorical characteristics or by Kruskal-Wallis rank sum tests for continuous characteristics to assess if there were any group differences in baseline characteristics.

Among participants with follow-up smoking status available from both data sources, agreement between 6-month prolonged abstinence from smoking status by source (survey versus HF) was calculated overall, then also by various date ranges, site, and subgroups of clinical interest. Positive predictive value (PPV), negative predictive value (NPV), sensitivity, specificity, and the Kappa statistic with 95% confidence interval were also calculated. PPV and sensitivity was calculated in reference to correctly identifying a quitter, and NPV and specificity was calculated in reference to correctly identifying an ongoing smoker. A Kappa value of 0.4–0.6 was considered moderately good agreement, and a Kappa of > 0.6 was considered good agreement. McNemar test was used to test if the marginal proportions of quitters from either source are equivalent or not.

To estimate the standard errors of PPV, NPV, sensitivity, and specificity we used a first order Taylor expansion approximation for the variance of the ratio of random variables, since the denominator of these two probabilities is also random [18]. To see if treatment had any effect on the main results, we repeated our main analyses within each treatment arm separately. The analysis for this paper was conducted by using SAS/STAT software, Version 9.2, by two members of the statistical core of the Center for Chronic Disease Outcomes Research (See author byline).

## Results

**Fig. 1:**
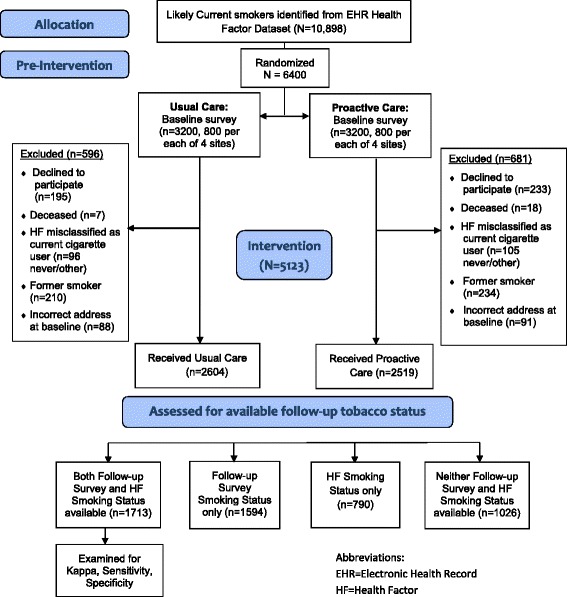
Results of randomization and availability of health factors

6400 participants were randomly selected and assigned to either proactive care or usual care and mailed a baseline survey to confirm smoking status. 1277 subjects were excluded for not meeting eligibility criteria: 428 declined to participate; 201 were misclassified as cigarette smokers (had never used cigarettes or smokeless tobacco user); 444 were former smokers (rather than current smokers) at the time of contact; 179 had incorrect mailing addresses; and 25 were deceased (Fig. 1).

Among the 5123 participants randomized and eligible for the original study, 33.5% (*n* = 1713) had both follow-up survey and HF data, 31.1% (*n* = 1594) had follow-up survey data only, 15.4% (*n* = 790) had HF data only, and 20.0% (*n* = 1026) had neither source of data. There were differences in participant baseline characteristics by data completion status. For instance, participants with neither source of data had the youngest median age (Both: 59, Survey: 60, HF: 56, Neither: 55; *p* < .001), least likely to be white (Both: 64.5%, Survey: 52.9%, HF: 61.8%, Neither: 52.1%; *p* < .001), and had the lowest median Charlson comorbidity index (Both: 1.0, Survey: 1.0, HF: 1.0, Neither: 0.0; *p* < .001 (Table 1).

**Table 1:** Baseline Characteristics of Participants in the Victory Trial by Source(s) of Follow-up Smoking Status (*N* = 5123). Values presented are n (%) or mean, tandard deviation and median (interquartile range) for continuous variables

Variable						p-value^1^
	Full Study Population *N* = 5123	Both Follow-up Survey and Health Factor Data *N* = 1713	Follow-Up Survey Data Only *N* = 1594	Health Factor Data Only *N* = 790	Neither Follow-up Survey nor Health Factor Data *N* = 1026	
Age in years	56.2 ± 12.0, 58 (12)	58.1 ± 9.6, 59 (11)	57.5 ± 11.5, 60 (13)	54.0 ± 12.6, 56 (15)	52.5 ± 14.5, 55 (19)	<.001
Age ≥ 65 years old	1094 (21.4%)	374 (21.8%)	402 (25.2%)	134 (17.0%)	184 (17.9%)	<.001
Male gender	4830 (94.3%)	1613 (94.2%)	1518 (95.2%)	741 (93.8%)	958 (93.4%)	.201
Race						<.001
White Black Other	2970 (58.0%) 1418 (27.7%) 735 (14.4%)	1105 (64.5%) 421 (24.6%) 187 (10.9%)	843 (52.9%) 485 (30.4%) 266 (16.7%)	488 (61.8%) 219 (27.7%) 83 (10.5%)	534 (52.1%) 293 (28.6%) 199 (19.4%)	
Smoking-related respiratory disease	960 (18.7%)	354 (20.7%)	294 (18.4%)	134 (17.0%)	178 (17.4%)	.066
Any mental illness	2465 (48.1%)	789 (46.1%)	736 (46.2%)	403 (51.0%)	537 (52.3%)	.002
Breakdown of diagnoses out of those with at least 1 mental illness:						
Depression PTSD Anxiety Substance Use Disorder Serious Mental Illness Other	1124 (45.6%) 466 (18.9%) 511 (20.7%) 1029 (41.7%) 367 (14.9%) 869 (35.3%)	347 (44.0%) 123 (15.6%) 177 (22.4%) 330 (41.8%) 111 (14.1%) 284 (36.0%)	338 (45.9%) 154 (20.9%) 119 (16.2%) 325 (44.2%) 104 (14.1%) 243 (33.0%)	175 (43.4%) 77 (19.1%) 95 (23.6%) 157 (39.0%) 76 (18.9%) 159 (39.5%)	264 (49.2%) 112 (20.9%) 120 (22.4%) 217 (40.4%) 76 (14.2%) 183 (34.1%)	.223 .030 .004 .328 .112 .155
Hospitalized in the past year	543 (10.6%)	188 (11.0%)	142 (8.9%)	104 (13.2%)	109 (10.6%)	.014
Number of hospitalizations in the prior year out of those with at least 1	1.67 ± 1.39, 1.00 (1.00)	1.52 ± 0.95, 1.00 (1.00)	1.56 ± 1.05, 1.00 (1.00)	1.76 ± 1.29, 1.00 (1.00)	1.97 ± 2.23, 1.00 (1.00)	0.541
Charlson comorbidity index	1.03 ± 1.44, 1.00 (2.00)	1.09 ± 1.42, 1.00 (2.00)	0.99 ± 1.38, 1.00 (1.00)	1.09 ± 1.52, 1.00 (2.00)	0.97 ± 1.52, 0.00 (1.00)	<.001
Site						<.001
A	1173 (22.9%)	620 (36.2%)	107 (6.7%)	336 (42.5%)	110 (10.7%)	
B	1384 (27.0%)	492 (28.7%)	447 (28.0%)	197 (24.9%)	248 (24.2%)	
C	1301 (25.4%)	22 (1.3%)	773 (48.5%)	25 (3.2%)	481 (46.9%)	
D	1265 (24.7%)	579 (33.8%)	267 (16.8%)	232 (29.4%)	187 (18.2%)	

Pearson Chi-square test was used for categorical variables and Kruskal-Wallis rank sum test was used for continuous variables to assess if there were any group differences

When examining differences between responders and nonresponders to the survey, responders were older (57.8 vs 53.2), and less likely to have mental illness (46.1% vs 51.8%). There were minor differences in response rates by site. When examining differences between subjects with HFs available and those without, subjects with health factors were more likely to be white (63.6% vs 52.6%) and more likely to have been hospitalized in the past year (11.7% vs 9.6%). There were large differences in availability of HFs by site. These analyses are available in Additional file 2.

### Agreement and availability by timeframe of HFs (Table 2)

For the full sample (1713) with HF and survey data identified within the full time frame examined (i.e., within 120 days of the follow-up survey mailing), sensitivity was 54.4%, specificity was 94.3%, Kappa was 0.48, and there was 90% smoking status agreement. Sensitivity, specificity, Kappa, and percent agreement remained relatively similar as the time interval between follow-up survey and HF data became shorter, though there was a modest drop off in sensitivity (5%) when compared to the full sample. A narrower timeframe resulted in an expected large drop off in the availability of HFs. Results of PPV and NPV were very similar to sensitivity and specificity. When we repeated these analyses stratified by treatment group, sensitivity, Kappa, and PPV were generally higher for the proactive arm than usual care. Agreement and NPV was slightly lower for the proactive group than the usual care group. Results were mixed with McNemar test results and specificity between the two treatment groups. These analyses are available in Additional file 3.

**Table 2:** Among participants in the Victory Trial with both data sources available, agreement between 6 month prolonged abstinence from smoking by survey, and Health Factor data drawn from different time intervals, and different sites. (*n* = 1713)

	% Available by Data Source	% Quitter by Follow-up Survey	% Quitter by Health Factor	% Quitter concordant Health Factor and Survey	Sensitivity (95% CI)	Specificity (95% CI)	Kappa (95% CI)	% Agreement
Agreement by Date Range:								
Health Factor Data within +/− 120 days of Survey Mailing (*n* = 1713) (full population)	100%	10.6%	10.9%	5.8%	54.4 (41.1, 67.7)	94.3 (87.5, 100.0)	0.48 (0.41, 0.55)	90.0%
Health Factor Data within +/− 90 days of Survey Mailing (*n* = 1357)	79.2%	10.6%	10.8%	5.6%	52.8 (38.1, 67.4)	94.1 (86.5, 100.0)	0.47 (0.39,0.54)	89.8%
Health Factor Data within +/− 60 days of Survey Mailing (*n* = 951)	55.5%	10.3%	10.2%	5.1%	49.0 (32.1, 65.9)	94.3 (85.2, 100.0)	0.43 (0.34, 0.53)	89.6%
Health Factor Data within +/− 30 days of Survey Mailing (*n* = 548)	32.0%	9.3%	8.9%	4.6%	49.0 (25.6, 72.5)	95.2 (83.2, 100.0)	0.45 (0.32, 0.58)	90.9%
Agreement by Site:								
Site A (*n* = 620)	36.2%	11.9%	10.7%	5.2%	43.2 (25.3, 61.2)	93.8 (82.5–100.0)	0.39 (0.28, 0.50)	87.7%
Site B (*n* = 492)	28.7%	9.6%	13.8%	7.3%	76.6 (43.3, 100.0)	92.8 (80.4–100.0)	0.59 (0.47, 0.69)	91.3%
Site C (*n* = 22)	1.3%	9.1%	9.1%	9.1%	N/A	N/A	N/A	N/A
Site D (*n* = 579)	33.8%	10.2%	8.8%	5.0%	49.2 (27.3, 71.0)	95.8 (84.0, 100.0)	0.48 (0.36, 0.60)	91.0%

Note: Survey data is considered the gold standard for this calculation. Site C excluded from analysis of agreement due to low numbers with health factor data

### Agreement and availability by site (Table 2)

**Fig. 2:**
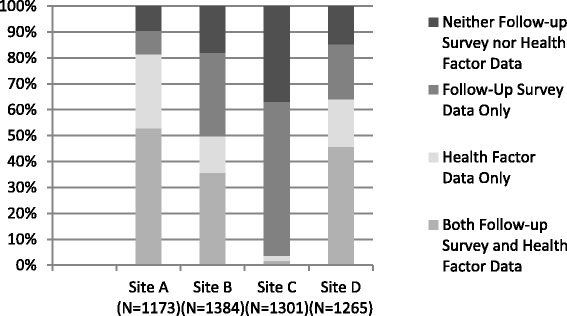
Variation in the availability of tobacco status by Health Factor, survey, both and neither by Site

Agreement differed by site with large variability in Kappa (Site A: 0.39, Site B: 0.59, Site C: excluded due to low numbers with HF data, Site D: 0.48) and sensitivity (Site A: 43.2, Site B: 76.6, Site C: excluded, Site D: 49.2) among the sites. There was also large variability in data completion status by site. Site A had a large proportion of patients with both sources of data (52.9%) and HF data overall (81.5%), and Site C had very few patients with both sources of data (1.7%) or HF data (3.6%) (Fig. 2).

### Key subgroups

Table 3 shows 1) agreement between follow-up survey smoking status and HF data smoking status and 2) smoking status for those with one source of data. Key subgroups included in this table are patients 65 years of age or older, patients with chronic lower respiratory disease, patients with any mental illness, and patients hospitalized in the past year. There was high agreement among patients who were 65 years of age or older (Sensitivity: 59.5, Specificity: 94.3, Kappa: 0.53, Agreement: 90.4%) and lower agreement among patients who were hospitalized in the past year (Sensitivity: 36.4, Specificity: 93.4, Kappa: 0.32, Agreement: 86.7%). In all of the subgroups, the population level quit rates identified via only survey or only HF data were higher than the population quit rates identified among patients with both sources of data.

**Table 3:** Agreement between follow-up survey and Health Factor data for 6 month prolonged abstinence from smoking among subgroups in the Victory Trial, as well as proportion of quitters for individuals with only one data source (*n* = 4097)

	Subjects with both Follow-up Survey and Health Factor Data (*n* = 1713)						Subjects with only one data source	
	% Quitter Follow-up Survey	% Quitter Health Factor	Sensitivity (95% CI)	Specificity (95% CI)	Kappa (95%CI)	% Agreement	Survey (*n* = 1594)	Health Factor (*n* = 790)
							Quitter n/ntot (%)	Quitter n/ntot (%)
Age ≥ 65 (*n* = 910)	11.9%	11.7%	59.5 (30.0, 89.0)	94.3 (79.7, 100.0)	0.53 (0.39, 0.66)	90.4%	56/402 (13.9%)	17/134 (12.7%)
Chronic Lower respiratory disease (*n* = 782)	9.3%	11.0%	54.5 (23.2, 85.9)	93.5 (78.7, 100.0)	0.44 (0.29, 0.60)	89.8%	39/294 (13.3%)	18/134 (13.4%)
Patients with any mental illness (*n* = 1928)	10.1%	9.9%	53.8 (33.8, 73.7)	95.1 (85.0, 100.0)	0.49 (0.39, 0.59)	90.9%	91/736 (12.4%)	56/403 (13.9%)
Patients hospitalized in the past year (*n* = 434)	14.0%	10.1%	36.4 (6.9, 65.8)	93.4 (72.9, 100.0)	0.32 (0.11–0.52)	86.7%	17/142 (12.0%)	11/104 (10.6%)

## Discussion

We compared the smoking cessation behaviors of participants in a randomized controlled trial, analyzing the agreement between survey methods and HFs drawn from the VA EHR. We found moderate agreement for quit behavior by Kappa statistics and sensitivity, but overall high concordance and similar population level quit rates. Though published data are few, our results are similar to the previous study comparing EHR and survey methods, in which the best agreement is found among continuous smokers and long-term non-smokers [11]. Although differences in the definition of smoking abstinence between survey and clinical assessments may account for some of the discrepancies in smoking status, we believe that the observed disagreement is predominantly due to the nature of tobacco cessation attempts. Recent quitters cycle in and out of attempts in relatively short periods of time, with most ultimately relapsing to tobacco use [19–21]. In this case, a discrepancy of a few weeks or months between when the HF and survey data were obtained would account for the difference. This is supported by the very high specificities and NPVs found, suggesting that for patients who remained smokers throughout, HFs are extremely accurate.

Despite these issues, the population level quit rates were very similar between the survey and HF methods. The observed discrepancies between methods of data collection may limit the validity of cessation data for individual patient-level inference, and researchers should be mindful of this when assessing the utility of HFs for answering a specific research question. However, the population rates of abstinence may yield very similar results when evaluated by either survey or HFs, and so HFs may be of greatest utility when this is the outcome of interest. The agreement in population level quit rates mean that HFs would likely provide a valid option for assessing population level abstinence or temporal changes as part of program evaluations, implementation studies, or pragmatic trials. They also provide a useful means for assessing epidemiologic trends in tobacco use, and examining outcomes of clinical innovations.

We did not find a decline in the agreement between HF and survey between wider and narrower timeframes. An even longer timeframe would likely increase availability, but we are unable to comment on the effect on accuracy. Site, more than any subgroup analyzed, had the greatest influence on the availability and accuracy of HFs, which is a major consideration for investigators considering using HFs in a research context. Similar to the HERC technical report [13], sites varied in the availability of follow-up HFs from over 80% of subjects to less than 5%. One site had more subjects with HF tobacco status available than subjects who responded to the survey, while the other three sites had fewer. The large variability in the availability of HFs by site may be related to differences in processes of who records these data, and how. For example, not all inpatient assessments of tobacco status generate HFs. Some HFs are standardized throughout the VA, but unique HFs can be created and used by any individual site. It is possible that some very uncommon HFs were used at a particular site that were not identified by our search algorithm, though we feel this is unlikely. For investigators considering using HFs for research or quality improvement purposes, a key first step would be assessing the availability at the institution in question.

We found some important demographic variation among patients who responded to the survey in comparison to those with HFs, as well as some variation in agreement among patient subgroups. Compared to survey responders, patients with HFs were younger, with higher rates of mental illness—two groups that may be contribute excessively to missing data gathered by survey. HFs are likely a more accurate means for filling in for non-response to survey than statistical methods, and can be employed in this capacity as an adjunct to survey administration. Nearly half of nonresponders to the survey had a HF status, and this number is likely to increase as HFs are employed more systematically throughout the VA [22]. We found that patients who were admitted to the hospital had the lowest agreement, though population level quit rates were again similar throughout. The low agreement for this group may be due to the fact that hospitalizations are a common time for a quit attempt [23]. Patients may indicate that they are a smoker during the admission, only to make a quit attempt shortly thereafter, which could have been captured by the survey. The highest agreement was found among patients over 65 years of age. Importantly, there was no difference in accuracy among patients with mental health diagnoses—a group with high smoking rates that is a priority for cessation interventions [24]. There were some moderate differences in population level quit rates between subjects with both sources of data vs only one, in which subjects with only survey or HF data tended to report a slightly higher quit rate. This suggests that there may be some unmeasured differences between patients who are nonresponders to surveys or are not captured by routine healthcare assessments.

Our study has some limitations. HF queries are made as part of face-to-face clinical interactions, and are not collected according to strict research definitions of tobacco abstinence, rather focusing on current smoking status only. HFs more often assess tobacco use as a whole, not just cigarette smoking. It is possible that patients who were dual users of both combustible and noncombustible tobacco may have been misclassified, as HFs may not allow capture of both forms of tobacco use. We do not have information on site and provider specific differences in how these queries were made that might explain the large variation by site. We compared the HF smoking status to smoking status by survey as our gold standard. While generally accurate [25], survey data may also be subject to inaccurate reporting, and we did see some variation in Kappa by study arm. This may be related to variations in how data was collected, or in recall bias on the part of patients who were or were not participating in a tobacco cessation program. Finally, these data refer to a Veteran population, that was largely white and male. This may limit the generalizability outside of the VA healthcare system, as both the EHR and patient population may not be representative of the US as a whole. However, as the largest single healthcare organization in the US, the data are likely to be applicable to other managed care organizations.

## Conclusions

In summary, we found that HFs provide a reasonably accurate and available means to measure change in smoking status over time among current smokers at baseline using the EHR, though their utility is strongly dependent on site. A majority of patients had a follow up tobacco status available in a similar timeframe to a 12 month survey, with substantial agreement with population level quit rates as determined by surveys or by HFs. The specificity for sustained smokers was very high. HFs appear to be a valid alternative for assessing quit behaviors on an administrative level, and could be used both as a primary measure of quit behaviors in the context of tobacco research, as a secondary source of data for patients who do not respond to surveys and lastly, as a source of data to examine epidemiologic trends within the VA.

## Additional files

Additional File 1: Methods for Determining Health Factor Status (DOCX 14 kb)

Additional File 2: Comparison of baseline characteristics of follow-up survey smoking status responders vs non-responders, and Comparison of baseline characteristics of follow-up HF smoking status availability vs not (DOCX 22 kb)

Additional File 3 Among participants in the Victory Trial with both data sources available, agreement between 6 month prolonged abstinence from smoking by survey, and Health Factor data drawn from different time intervals, and different sites; stratified by treatment arm. (DOCX 55 kb)

## Abbreviations

EHR: Electronic Health Record
HERC: Health Economic Resource Center
HF: Health Factor
ICD-9: International Classification of Diseases, 9th Edition
IRB: Institutional Review Board
NPV: Negative Predictive Value
PPV: Positive Predictive Value
PTSD: Post Traumatic Stress Disorder
RCT: Randomized Controlled Trial
VA: Department of Veterans Affairs
VICTORY: Veterans Victory Over Tobacco Trial
