# Supplementary material for: Validating the use of veterans affairs tobacco health factors for assessing change in smoking status: accuracy, availability, and approach
Source: BMC Med Res Methodol. 2018 May 11;18:39. doi: 10.1186/s12874-018-0501-2 (PMC5948734; doi:10.1186/s12874-018-0501-2)
Supplement: Supplementary file 1 — Methods for Determining Health Factor Status (DOCX 14 kb) [file 12874_2018_501_MOESM1_ESM.docx]

Additional File 1:

**Methods for Determining Health Factor Status:**

Health Factor data was extracted from the VA medical record (CPRS) using the following methods:

A program was written to pull health factors for each patient seen at the sites participating in the study. Given that patients frequently transfer care between nearby sites, particularly tertiary care centers, the program also drew health factors from neighboring VA clinics or hospitals where the patient received care. For the purposes of recruitment, patients were categorized for invitation to participate in the study as “Current Smoker,” “Recent quitter” “Quitter, greater than 1 year,” “Other tobacco user” or “Non tobacco user. Based on these results, subjects were considered for inclusion in the study. The follow-up tobacco status was determined by using these same categories at +/- 120 days of follow-up date. A change in status from “current smoker” to any category indicating no current use of tobacco was assigned a status of quitter.

The specific health factors and their assignments were as follows:

1. “Current Smoker” if health factor label contained : ‘CURRENT SMOKER’, ’TOBACCO CURRENT USER’, ‘CURRENTLY USES TOBACCO’, ‘DOES NOT WANT TO QUIT SMOKING’, ‘PATIENT IS TOBACCO USER’, ‘TOBACCO PATIENT REFUSES TO QUIT’, ‘READY TO QUIT TOBACCO USE’, ‘REFUSED SMOKING CESSATION’, ‘PATIENT IS SMOKER’, ‘SMOKED IN PAST YEAR’, ‘THINKING ABOUT QUIT TOBACCO USE’, ‘TOBACCO USER IN PAST YEAR’, or ‘TOBACCO USER’.
2. “Quit within past year” if the heath factor label contained: 'QUIT TOBACCO IN THE LAST 12 MONTHS', 'NON TOBACCO USER - QUIT IN PAST YEAR', 'FORMER TOBACCO USE <1Y', 'QUIT SMOKING WITHIN PAST YEAR', 'IN LAST YEAR QUIT TOBACCO USE', 'NO TOBACCO < 12M', 'NOT CURRENT SMOKER', 'QUIT SMOKING WITHIN PAST YEAR', 'QUIT TOBACCO USE IN LAST YEAR', 'TOBACCO FORMER USER LESS 12 MONTHS', 'TOBACCO NON USE LESS THAN 12 MONTHS', 'TOBACCO: NON-USER <= 12 MONTHS', 'CESSATION < 12 MONTHS', 'TOBACCO QUIT <12 MO', or ‘NOT CURRENT SMOKER’.
3. “Quit more than one year” if the heath factor label contained: 'FORMER USER MORE 12 MONTHS', 'FORMER TOBACCO USE >1Y <7Y', 'FORMER TOBACCO USER 7Y OR GREATER', 'FORMER TOBACCO USER: QUIT<7 YEARS AGO', 'NON TOBACCO USER - QUIT >1 YEAR AGO', 'NON TOBACCO USER - QUIT >7 YEARS AGO', 'QUIT SMOKING > 1YR', 'QUIT SMOKING MORE THAN 12 MONTHS', 'QUIT TOBACCO >12 MO & <7 YRS AGO', 'QUIT TOBACCO >7 YEARS AGO', 'QUIT TOBACCO USE > 7 YEARS AGO', 'TOB QUIT TOBACCO >7 YEARS AGO', 'TOBACCO FORMER USER MORE 12 MONTHS', 'TOBACCO NON USE GREATER THAN 12 MONTHS', 'TOBACCO: NON-USER >12 MONTHS', 'TOBACCO CESSATION <7 YEARS', 'TOBACCO CESSATION > 12 MONTHS', or 'TOBACCO USE NONE IN > 7 YEARS'.
4. “Other Tobacco User” if the heath factor label contained: 'SMOKELESS TOBACCO USER', 'CURRENTLY CHEWS TOBACCO', 'TOBACCO CHEW USER', or 'TOBACCO CIGAR SMOKER'.
5. “Non Tobacco User” if the heath factor label contained: 'LIFETIME NON-SMOKER', 'LIFETIME NON-TOBACCO USER', 'LIFETIME NON-USER OF TOBACCO', 'TOBACCO NON-USER', 'CURRENT NON-TOBACCO USER', 'NON-TOBACCO USER', or 'TOBACCO LIFELONG NON USER'.
