# Supplementary material for: Validating the use of veterans affairs tobacco health factors for assessing change in smoking status: accuracy, availability, and approach
Source: BMC Med Res Methodol. 2018 May 11;18:39. doi: 10.1186/s12874-018-0501-2 (PMC5948734; doi:10.1186/s12874-018-0501-2)
Supplement: Supplementary file 2 — Comparison of baseline characteristics of follow-up survey smoking status responders vs non-responders, and Comparison of baseline characteristics of follow-up HF smoking status availability vs not (DOCX 22 kb) [file 12874_2018_501_MOESM2_ESM.docx]

| Additional Table 1. Comparison of baseline characteristics of follow-up survey smoking status responders vs non-responders. | | | | |
| --- | --- | --- | --- | --- |
| Characteristic | Full Study Population N=5123 | Responders n=3307 | Non-responders n=1816 | p-value^1^ |
| Age in years, mean (SD) | 56.2 (12.0) | 57.8 (10.5) | 53.2 (13.7) | <0.001 |
| Age ≥ 65 years old, n (%) | 1094 (21.4%) | 776 (23.5%) | 318 (17.5%) | <0.001 |
| Male gender, n (%) | 4830 (94.3%) | 3131 (94.7%) | 1699 (93.6%) | 0.099 |
| Race, n (%)  White  Black  Other | 2970 (58.0%)  1418 (27.7%)  735 (14.4%) | 1948 (58.9%)  906 (27.4%)  453 (13.7%) | 1022 (56.3%)  512 (28.2%)  282 (15.5%) | 0.111 |
| Chronic Lower respiratory disease, n (%) | 960 (18.7%) | 648 (19.6%) | 312 (17.2%) | 0.034 |
| Any mental illness, n (%) | 2465 (48.1%) | 1525 (46.1%) | 940 (51.8%) | <0.001 |
| Breakdown of diagnoses out of those with at least 1 mental illness, n (%): |  |  |  |  |
| Depression | 1124 (45.6%) | 685 (44.9%) | 439 (46.7%) | 0.388 |
| PTSD | 466 (18.9%) | 277 (18.2%) | 189 (20.1%) | 0.232 |
| Anxiety | 511 (20.7%) | 296 (19.4%) | 215 (22.9%) | 0.039 |
| Substance Use Disorder | 1029 (41.7%) | 655 (43.0%) | 374 (39.8%) | 0.122 |
| Serious MI | 367 (14.9%) | 215 (14.1%) | 152 (16.2%) | 0.161 |
| Other | 869 (35.3%) | 527 (34.6%) | 342 (36.4%) | 0.357 |
| Hospitalized in the past year, n (%) | 543 (10.6%) | 330 (10.0%) | 213 (11.7%) | 0.052 |
| Number of hospitalizations in the prior year out of those with at least 1, mean (SD) | 1.67 (1.39) | 1.54 (1.00) | 1.87 (1.83) | 0.194 |
| Charlson comorbidity index, mean (SD) | 1.03 (1.44) | 1.04 (1.40) | 1.02 (1.52) | 0.028 |
| Site, n (%) |  |  |  | <0.001 |
| A | 1173 (22.9%) | 727 (22.0%) | 446 (24.6%) |  |
| B | 1384 (27.0%) | 939 (28.4%) | 445 (24.5%) |  |
| C | 1301 (25.4%) | 795 (24.0%) | 506 (27.9%) |  |
| D | 1265 (24.7%) | 846 (25.6%) | 1. 3.1%) |  |

^1^Pearson Chi-square test was used for categorical variables and Kruskal-Wallis rank sum test was used for continuous variables to assess if there were any group differences.

| Additional Table 2: Comparison of baseline characteristics of follow-up HF smoking status availability vs not (NOT taking into account the complex study design). | | | | |
| --- | --- | --- | --- | --- |
| Characteristic | Full Study Population N=5123 | Available n=2503 | Not Available n=2620 | p-value^1^ |
| Age in years, mean (SD) | 56.2 (12.0) | 56.8 (10.8) | 55.5 (13.0) | 0.075 |
| Age ≥ 65 years old, n (%) | 1094 (21.4%) | 508 (20.3%) | 586 (22.4%) | 0.071 |
| Male gender, n (%) | 4830 (94.3%) | 2354 (94.1%) | 2476 (94.5%) | 0.482 |
| Race, n (%)  White  Black  Other | 2970 (58.0%)  1418 (27.7%)  735 (14.4%) | 1593 (63.6%)  640 (25.6%)  270 (10.8%) | 1377 (52.6%)  778 (29.7%)  465 (17.8%) | <0.001 |
| Chronic Lower respiratory disease, n (%) | 960 (18.7%) | 488 (19.5%) | 472 (18.0%) | 0.174 |
| Any mental illness, n (%) | 2465 (48.1%) | 1192 (47.6%) | 1273 (48.6%) | 0.490 |
| Breakdown of diagnoses out of those with at least 1 mental illness, n (%): |  |  |  |  |
| Depression | 1124 (45.6%) | 522 (43.8%) | 602 (47.3%) | 0.081 |
| PTSD | 466 (18.9%) | 200 (16.8%) | 266 (20.9%) | 0.009 |
| Anxiety | 511 (20.7%) | 272 (22.8%) | 239 (18.8%) | 0.013 |
| Substance Use Disorder | 1029 (41.7%) | 487 (40.9%) | 542 (42.6%) | 0.387 |
| Serious MI | 367 (14.9%) | 187 (15.7%) | 180 (14.1%) | 0.281 |
| Other | 869 (35.3%) | 443 (37.2%) | 426 (33.5%) | 0.055 |
| Hospitalized in the past year, n (%) | 543 (10.6%) | 292 (11.7%) | 251 (9.6%) | 0.015 |
| Number of hospitalizations in the prior year out of those with at least 1, mean (SD) | 1.67 (1.39) | 1.61 (1.09) | 1.74 (1.68) | 0.894 |
| Charlson comorbidity index, mean (SD) | 1.03 (1.44) | 1.09 (1.45) | 0.98 (1.44) | <0.001 |
| Site, n (%) |  |  |  | <0.001 |
| A | 1173 (22.9%) | 956 (38.2%) | 217 (8.3%) |  |
| B | 1384 (27.0%) | 689 (27.5%) | 695 (26.5%) |  |
| C | 1301 (25.4%) | 47 (1.9%) | 1254 (47.9%) |  |
| D | 1265 (24.7%) | 811 (32.4%) | 1. 7.3%) |  |

^1^Pearson Chi-square test was used for categorical variables and Kruskal-Wallis rank sum test was used for continuous variables to assess if there were any group differences.
