# Supplementary material for: Validating the use of veterans affairs tobacco health factors for assessing change in smoking status: accuracy, availability, and approach
Source: BMC Med Res Methodol. 2018 May 11;18:39. doi: 10.1186/s12874-018-0501-2 (PMC5948734; doi:10.1186/s12874-018-0501-2)
Supplement: Supplementary file 3 — Among participants in the Victory Trial with both data sources available, agreement between 6 month prolonged abstinence from smoking by survey, and Health Factor data drawn from different time intervals, and different sites; stratified by treatment arm. (DOCX 55 kb) [file 12874_2018_501_MOESM3_ESM.docx]

| Additional File 3:  Among participants in the Victory Trial with both data sources available, agreement between 6 month prolonged abstinence from smoking by survey, and Health Factor data drawn from different time intervals, and different sites; stratified by treatment arm.  Additional Table 3a: Among participants in the Victory Trial with both data sources available, agreement between 6 month prolonged abstinence from smoking by survey, and Health Factor data drawn from different time intervals, and different sites, (n=1713) | | | | | | | | | | | | |
| --- | --- | --- | --- | --- | --- | --- | --- | --- | --- | --- | --- | --- |
|  |  | **% Quitter by Follow-up Survey** | **% Quitter by Health Factor** | **McNemar test result** | **% Quitter by both Health Factor and Survey** | **Sensitivity (95% CI)** | **Specificity (95% CI)** | **Kappa (95% CI)** | **% Agreement** | **PPV (95% CI)** | **NPV (95% CI)** | |
|  | **% Available by Data Source** |  |  |  |  |  |  |  |  |  |  |  |
| Agreement by Date Range: | | | | | | | | | |  |  | |
| Health Factor Data within +/- 120 days of Survey Mailing (n=1713)  (full population) |  | 10.6% | 10.9% | S=0.15, p=0.702 | 5.8% | 54.4  (41.1, 67.7) | 94.3  (87.5, 100.0) | 0.48  (0.41, 0.55) | 90.0% | 52.9 (40.0, 65.8) | 94.6 (87.8, 100.0) | |
|  | 100% |  |  |  |  |  |  |  |  |  |  |  |
| Health Factor Data within +/- 90 days of Survey Mailing (n=1357) |  | 10.6% | 10.8% | S=0.06, p=0.799 | 5.6% | 52.8  (38.1, 67.4) | 94.1  (86.5, 100.0) | 0.47  (0.39,0.54) | 89.8% | 51.7 (37.4, 66.0) | 94.4 (86.7, 100.0) | |
|  | 79.2% |  |  |  |  |  |  |  |  |  |  |  |
| Health Factor Data within +/- 60 days of Survey Mailing (n=951) |  | 10.3% | 10.2% | S=0.01, p=0.920 | 5.1% | 49.0  (32.1, 65.9) | 94.3  (85.2, 100.0) | 0.43  (0.34, 0.53) | 89.6% | 49.5 (32.4, 66.6) | 94.1 (85.0, 100.0) | |
|  | 55.5% |  |  |  |  |  |  |  |  |  |  |  |
| Health Factor Data within +/- 30 days of Survey Mailing (n=548) | 32.0% | 9.3% | 8.9% | S=0.08, p=0.777 | 4.6% | 49.0  (25.6, 72.5) | 95.2  (83.2, 100.0) | 0.45  (0.32, 0.58) | 90.9% | 51.0 (26.4, 75.6) | 94.8 (82.9, 100.0) | |
| Agreement by Site: | | | | | | | | | |  |  | |
| **Site A (n=620)** | 36.2% | 11.9% | 10.7% | S=0.84, p=0.359 | 5.2% | 43.2  (25.3, 61.2) | 93.8  (82.5-100.0) | 0.39  (0.28, 0.50) | 87.7% | 48.5 (28.0, 69.0) | 92.4 (81.3, 100.0) | |
| **Site B (n=492)** | 28.7% | 9.6% | 13.8% | S=10.26, **p=0.001** | 7.3% | 76.6   (43.3, 100.0) | 92.8  (80.4-100.0) | 0.59  (0.47, 0.69) | 91.3% | 52.9 (31.6, 74.3) | 97.4 (84.2, 100.0) | |
| **Site C (n=22)** | 1.3% | 9.1% | 9.1% | N/A | 9.1% | N/A | N/A | N/A | N/A | N/A | N/A | |
| **Site D (n=579)** | 33.8% | 10.2% | 8.8% | S=1.23, p=0.267 | 5.0% | 49.2  (27.3, 71.0) | 95.8  (84.0, 100.0) | 0.48  (0.36, 0.60) | 91.0% | 56.9 (30.9, 82.8) | 94.3 (82.8, 100.0) | |
| Note: Survey data is considered the gold standard for this calculation. Site C excluded from analysis of agreement due to low numbers with health factor data. | | | | | | | | | |  |  | |

| Additional Table 3b. Among participants in the Victory Trial with both data sources available, agreement between 6 month prolonged abstinence from smoking by survey, and Health Factor data drawn from different time intervals, and different sites. (n=793, Proactive Only) | | | | | | | | | | | | |
| --- | --- | --- | --- | --- | --- | --- | --- | --- | --- | --- | --- | --- |
|  |  | **% Quitter by Follow-up Survey** | **% Quitter by Health Factor** | **McNemar test result** | **% Quitter by both Health Factor and Survey** | **Sensitivity (95% CI)** | **Specificity (95% CI)** | **Kappa (95% CI)** | **% Agreement** | **PPV (95% CI)** | **NPV (95% CI)** | |
|  | **% Available by Data Source** |  |  |  |  |  |  |  |  |  |  |  |
| Agreement by Date Range: | | | | | | | | | |  |  | |
| Health Factor Data within +/- 120 days of Survey Mailing (n=793)  (full population) |  | 12.2% | 12.2% | S=0.00, p=1.000 | 7.1% | 57.7  (38.7, 76.7) | 94.1  (84.1, 100.0) | 0.52  (0.43, 0.61) | 89.6% | 57.7 (38.7, 76.7) | 94.1 (84.1, 100.0) | |
|  | 100% |  |  |  |  |  |  |  |  |  |  |  |
| Health Factor Data within +/- 90 days of Survey Mailing (n=638) |  | 12.7% | 12.9% | S=0.01, p=0.906 | 7.2% | 56.8  (36.2, 77.3) | 93.5  (82.4, 100.0) | 0.50  (0.40,0.60) | 88.9% | 56.1 (35.8, 76.4) | 93.7 (82.5, 100.0) | |
|  | 80.5% |  |  |  |  |  |  |  |  |  |  |  |
| Health Factor Data within +/- 60 days of Survey Mailing (n=440) |  | 11.6% | 12.1% | S=0.08, p=0.773 | 6.4% | 54.9  (29.6, 80.2) | 93.6  (80.2, 100.0) | 0.48  (0.35, 0.60) | 89.1% | 52.8 (28.6, 77.0) | 94.1 (80.6, 100.0) | |
|  | 55.5% |  |  |  |  |  |  |  |  |  |  |  |
| Health Factor Data within +/- 30 days of Survey Mailing (n=260) | 32.9% | 11.5% | 10.0% | S=0.67, p=0.414 | 6.2% | 53.3  (21.0, 85.7) | 95.7  (78.0, 100.0) | 0.52  (0.35, 0.69) | 90.8% | 61.5 (23.2, 99.9) | 94.0 (76.7, 100.0) | |
| Agreement by Site: | | | | | | | | | |  |  | |
| **Site A (n=280)** | 35.3% | 12.9% | 11.4% | S=0.50, p=0.480 | 6.4% | 50.0  (21.7, 78.3) | 94.3  (77.3-100.0) | 0.46  (0.31, 0.62) | 88.6% | 56.3 (23.8, 88.7) | 92.7 (76.1, 100.0) | |
| **Site B (n=236)** | 29.8% | 10.2% | 15.3% | S=7.20, **p=0.007** | 8.5% | 83.3   (33.9, 100.0) | 92.5  (74.5-100.0) | 0.62  (0.47, 0.77) | 91.5% | 55.6 (25.2, 85.9) | 98.0 (78.7, 100.0) | |
| **Site C (n=7)** | 0.9% | 28.6% | 28.6% | N/A | 28.6% | N/A | N/A | N/A | N/A | N/A | N/A | |
| **Site D (n=270)** | 34.0% | 13.0% | 10.0% | S=2.13, p=0.144 | 5.9% | 45.7  (18.7, 72.8) | 95.3 (77.9, 100.0) | 0.45  (0.29, 0.62) | 88.9% | 59.3 (22.6, 95.9) | 92.2 (75.4, 100.0) | |
| Note: Survey data is considered the gold standard for this calculation. Site C excluded from analysis of agreement due to low numbers with health factor data. | | | | | | | | | | | | |

| Additional Table 3c. Among participants in the Victory Trial with both data sources available, agreement between 6 month prolonged abstinence from smoking by survey, and Health Factor data drawn from different time intervals, and different sites. (n=920, Usual Care Only) | | | | | | | | | | | | |
| --- | --- | --- | --- | --- | --- | --- | --- | --- | --- | --- | --- | --- |
|  |  | **% Quitter by Follow-up Survey** | **% Quitter by Health Factor** | **McNemar test result** | **% Quitter by both Health Factor and Survey** | **Sensitivity (95% CI)** | **Specificity (95% CI)** | **Kappa (95% CI)** | **% Agreement** | **PPV (95% CI)** | **NPV (95% CI)** | |
|  | **% Available by Data Source** |  |  |  |  |  |  |  |  |  |  |  |
| Agreement by Date Range: | | | | | | | | | |  |  | |
| Health Factor Data within +/- 120 days of Survey Mailing (n=920)  (full population) |  | 9.2% | 9.8% | S=0.28, p=0.596 | 4.7% | 50.6  (32.0, 69.1) | 94.4  (85.2, 100.0) | 0.44  (0.34, 0.54) | 90.3% | 47.8 (30.4, 65.1) | 94.9 (85.7, 100.0) | |
|  | 100% |  |  |  |  |  |  |  |  |  |  |  |
| Health Factor Data within +/- 90 days of Survey Mailing (n=719) |  | 8.8% | 9.0% | S=0.06, p=0.808 | 4.2% | 47.6  (26.9, 68.3) | 94.7  (84.3, 100.0) | 0.42  (0.30,0.53) | 90.5% | 46.2 (26.2, 66.1) | 95.0 (84.5, 100.0) | |
|  | 78.2% |  |  |  |  |  |  |  |  |  |  |  |
| Health Factor Data within +/- 60 days of Survey Mailing (n=511) |  | 9.2% | 8.6% | S=0.18, p=0.674 | 3.9% | 42.6  (20.3, 64.8) | 94.8  (82.5, 100.0) | 0.38  (0.25, 0.52) | 90.0% | 45.5 (21.4, 69.5) | 94.2 (81.9, 100.0) | |
|  | 55.5% |  |  |  |  |  |  |  |  |  |  |  |
| Health Factor Data within +/- 30 days of Survey Mailing (n=288) | 31.3% | 7.3% | 8.0% | S=0.15, p=0.695 | 3.1% | 42.9  (9.4, 76.3) | 94.8  (78.5, 100.0) | 0.36  (0.17, 0.55) | 91.0% | 39.1 (9.0, 69.3) | 95.5 (79.0, 100.0) | |
| Agreement by Site: | | | | | | | | | |  |  | |
| **Site A (n=340)** | 37.0% | 11.2% | 10.0% | S=0.36, p=0.547 | 4.1% | 36.8  (14.3, 59.4) | 93.4  (78.2-100.0) | 0.32 (0.16, 0.47) | 87.1% | 41.2 (15.5, 66.8) | 92.2 (77.2, 100.0) | |
| **Site B (n=256)** | 27.8% | 9.0% | 12.5% | S=3.52, p=0.061 | 6.3% | 69.6   (25.2, 100.0) | 93.1 (75.9-100.0) | 0.53  (0.37, 0.70) | 91.0% | 50.0 (20.0, 80.0) | 96.9 (78.8, 100.0) | |
| **Site C (n=15)** | 1.6% | 0.0% | 0.0% | N/A | 0.0% | N/A | N/A | N/A | N/A | N/A | N/A | |
| **Site D (n=309)** | 33.6% | 7.8% | 7.8% | S=0.00m p=1.000 | 4.2% | 54.2  (17.6, 90.7) | 96.1  (80.2, 100.0) | 0.50  (0.32, 0.68) | 92.9% | 54.2 (17.6, 90.7) | 96.1 (80.2, 100.0) | |
| Note: Survey data is considered the gold standard for this calculation. Site C excluded from analysis of agreement due to low numbers with health factor data. | | | | | | | | | | | | |
